# Differences in HIV, Syphilis, and Hepatitis B Virus Screening Coverage between Pregnant Women Presenting to Antenatal Care in Early and Late Pregnancy in Indonesia

**DOI:** 10.21315/mjms-08-2025-608

**Published:** 2025-12-31

**Authors:** Mona Safitri Fatiah, Agung Dwi Laksono, Apriyana Irjayanti, Afwan Syarif

**Affiliations:** 1 Department of Reproductive Health, Faculty of Public Health, Cenderawasih University, Jayapura, Papua, Indonesia; 2 National Research and Innovation Agency, Jakarta, Indonesia; 3 Department of Environmental Health and Occupational Health, Faculty of Public Health, Cenderawasih University, Jayapura, Papua, Indonesia; 4 Department of Public Health, Budi Mulya College of Health Sciences, Sukajaya, Palembang, South Sumatra, Indonesia

**Keywords:** antenatal care, elimination of mother-to-child transmission, human immunodeficiency virus, syphilis, hepatitis B virus

## Abstract

**Background:**

Timely initiation of antenatal care (ANC), defined as the first ANC visit during the first trimester, is critical for the elimination of mother-to-child transmission (EMTCT) of human immunodeficiency virus (HIV), syphilis, and hepatitis B virus (HBV). In Indonesia, service coverage for these infections remains uneven, and the sociodemographic and service-related determinants of complete screening are not well understood.

**Methods:**

We analysed data from the 2023 Indonesian Health Survey in a cross-sectional study of pregnant women. Multivariable logistic regression was used to assess the effects of ANC initiation timing, maternal education, parity, and region on complete screening coverage, defined as receipt of all three recommended for HIV, syphilis, and HBV.

**Results:**

Most respondents were aged 20 to 34 years, and over half initiated ANC in the first trimester. Screening coverage exceeded 75% in Java, Nusa Tenggara, Kalimantan, and Papua, but was below 45% in parts of Sumatra and Eastern Indonesia. Late ANC initiation was associated with lower odds of complete screening (adjusted odds ratio [AOR]) = 0.68; 95% CI: 0.53, 0.86; *P* < 0.05). Regional disparities persisted after adjustment: women in Sumatra (AOR = 0.32; 95% CI: 0.25, 0.42), Sulawesi (AOR = 0.59; 95% CI: 0.45, 0.79), and Maluku-Papua (AOR = 0.47; 95% CI: 0.33, 0.69) had significantly lower uptake compared with Java-Bali. Lower educational attainment and high parity were also associated with reduced screening.

**Conclusion:**

Timing ANC initiation, maternal education, parity, and region of residence are strong determinants of comprehensive screening coverage for HIV, syphilis, and HBV among pregnant women. Policy efforts should focus on promoting early ANC attendance and reducing regional inequities to accelerate EMTCT progress in Indonesia.

## Introduction

The elimination of mother-to-child transmission (EMTCT) of human immunodeficiency virus (HIV), syphilis, and hepatitis B virus (HBV) is recognised by the World Health Organization (WHO) as a critical global public health priority for maternal and child health (1). Each year, approximately 120,000 infants acquire HIV, around 700,000 cases of congenital syphilis occur due to 1.1 million new maternal infections, and an estimated 100,000 childhood HBV infections arise from over one million maternal cases (2–5). Collectively, these preventable ailments contribute substantially to perinatal morbidity, mortality, and long-term chronic paediatric diseases. To address this burden, WHO promotes an integrated EMTCT framework that emphasises early antenatal care (ANC) attendance as a gateway for universal screening, timely diagnosis, and treatment of HIV, syphilis, and HBV among pregnant women (6). Early ANC initiation (within the first trimester) is critical for identifying infections early enough to initiate treatment and prevent vertical transmission, whereas late ANC presentation (second or third trimester) often results in missed opportunities for screening or delayed management (7, 8). This timing gap is therefore pivotal to EMTCT outcomes and forms the core focus of the present analysis.

In the Asia-Pacific region, the United Nations Children’s Fund (UNICEF)–WHO Regional Roadmap (2024–2030) seeks accelerated EMTCT through universal health coverage, integrated service delivery, and equity-oriented approaches (7). Accordingly, the Indonesia Ministry of Health introduced the *National EMTCT Guideline* in 2018, recommending universal screening for HIV, syphilis, and HBV at the first ANC visit, with a national target of 95% screening coverage by 2030 (8). However, implementation has been uneven, with significant regional and facility-level disparities in test availability, supply chain stability, and healthcare provider adherence to guidelines. According to the National HIV/Acquired Immune Deficiency Syndrome Information System AIDS (SIHA) for 2023–2024, screening coverage remains below target: HIV testing reached 83%, syphilis 64%, and HBV 58% among pregnant women attending ANC (9, 10). Local studies further highlight missed opportunities, as only a small proportion of women testing positive for HIV or syphilis received appropriate treatment or follow-up (11). Additionally, sentinel ANC surveillance is geographically limited and often delayed, constraining national programme evaluation (11, 12).

Most existing studies in Indonesian report aggregate EMTCT screening data without distinguishing between women initiating ANC early and those presenting later in pregnancy. This limits understanding of when, during pregnancy, missed screening opportunities occur. Addressing this evidence gap, the present study examines differences in HIV, syphilis, and HBV screening coverage between pregnant women presenting to ANC services in early and late pregnancy in Indonesia, while also assessing maternal and regional determinants of screening HBV uptake. These findings aim to inform targeted policy and programme interventions to strengthen timely screening and advance EMTCT goals nationwide.

## Methods

### Research Design

This study utilised data from the 2023 Indonesian Health Survey (Survei Kesehatan Indonesia, SKI 2023), accessed through an official data request to the Ministry of Health of the Republic of Indonesia (http://layanandata.kemkes.go.id). The SKI 2023 is a nationally representative, cross-sectional survey conducted by the Ministry of Health in collaboration with Statistics Indonesia (BPS), covering all provinces and districts/municipalities in Indonesia. Structured interviews were conducted with household members using standardised electronic questionnaires to collect demographic, health service, and maternal health data. Detailed information on the sampling design and data collection procedures is available in the SKI 2023 National Report (10).

### Setting and Samples

The target population consisted of 55,055 pregnant women interviewed in the SKI 2023 survey. After applying inclusion and exclusion criteria, the final analytic sample included 1,963 women who had valid and complete information on HIV, syphilis, and HBV screenings. Inclusion criteria were as follows: i) pregnant women who reported having undergone at least one of the three recommended screening tests (HIV, syphilis, or HBV) during pregnancy, whether at the first antenatal contact (Trimester I) or subsequent visits (Trimester II–III); ii) women who had resided or intended to reside in the survey area for a minimum of six months consistent with the household eligibility criteria of SKI to ensure accurate representation of the community population; and iii) respondents who were able to recall and report the type of screening tests received during pregnancy and who provided informed consent to participate in the survey.

Women with missing data on all screening indicators and those residing in conflict-affected areas were excluded. The exclusion of these groups may have led to a slight underestimation of national coverage and is acknowledged as a limitation. The SKI 2023 employed a multistage cluster random sampling design. Census blocks served as the primary sampling unit (PSU), selected from the national sampling frame maintained by BPS. Within each PSU, households were selected using systematic random sampling, and all eligible women were interviewed. Sampling weights provided in the dataset were applied to consider complex survey design, adjust for nonresponse, and ensure national representativeness. The reported household response rate was 91.4%.

### Measurement and Data Collection

#### Outcome Variable

The main outcome was the completeness of HIV, syphilis, and HBV screening during pregnancy, coded as 0 = Incomplete (one or two tests only or none) and 1 = Complete (all three tests performed). Although only women who reported at least one test were included, the completeness variable allowed identification of partial versus full screening coverage. Screening information was self-reported by respondents during interviews and cross-checked against maternal health cards when available.

#### Exposure Variables

The primary exposure was the timing of the first ANC visit, categorised as early pregnancy (≤ 13 weeks, first trimester) and late pregnancy (≥ 14 weeks, second or third trimester), in accordance with WHO recommendations for early ANC initiation before 12 to 13 weeks.

#### Control Variables

Covariates included maternal age (20 to 34 years vs. < 20 or ≥ 35 years); maternal education (college, senior high school, junior high school, or primary school); place of residence (urban or rural); region (Java-Bali, Sumatra, Kalimantan, Sulawesi, Nusa Tenggara, or Papua-Maluku); age at first pregnancy (20 to 34 years vs. < 19 or ≥ 35 years); gravidity (multigravida 2–4, primigravida, or grand multipara ≥ 5); parity (multipara 2–4, primipara/nullipara, or grand multipara ≥ 5); pregnancy intention (intended or unintended); ANC provider (obstetrician, general practitioner, midwife, or other health worker); and place of health check-up (primary healthcare, hospital, private clinic/practice, or integrated/village maternity post).

### Data Analysis

All analyses were performed using Stata version 17 (StataCorp, College Station, TX, USA). Descriptive statistics summarised maternal and pregnancy characteristics, and group differences were examined using the chi-square test. Binary logistic regression model was applied to identify determinants with incomplete screening. Variables with theoretical importance (*P* < 0.25) in bivariate analysis were entered in the multivariate model using the enter method. Adjusted odds ratios (AOR) and 95% confidence intervals (CI) were reported. Multicollinearity was assessed using the variance inflation factor (VIF); variables with VIF > 10 were excluded. Model fit was tested with the Hosmer–Lemeshow goodness-of-fit statistic. Missing data for key variables were below 5% and randomly distributed; therefore, listwise deletion was applied as a valid approach without introducing bias.

### Spatial Analysis

Spatial analysis was conducted to visualise regional variations in screening coverage. Weighted prevalence estimates were aggregated by district/municipality level and mapped using ArcGIS version 10.8, generating choropleth maps for inter-regional comparisons.

### Ethical Considerations

The SKI 2023 was approved by the National Ethics Committee of the Indonesian Ministry of Health (LB.02.01/I/KE/L/287/2023). Informed consent was obtained from all participants prior to data collection. The anonymised dataset is publicly available for academic purposes through the data portal of the Ministry of Health (https://layanandata.kemkes.go.id/).

## Results

**Figure 1:**
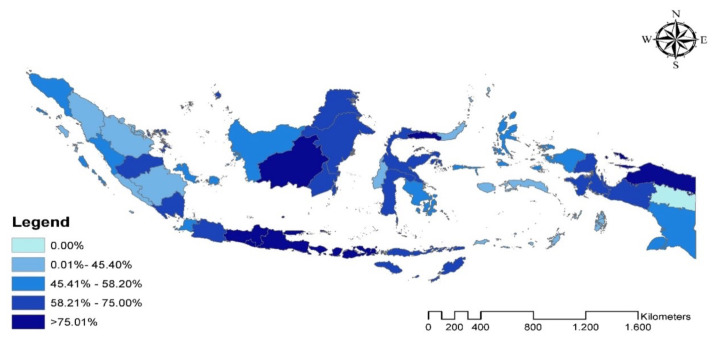
Comprehensive testing coverage (HIV, syphilis, and hepatitis B) among pregnant women by province, Indonesia, 2023

Figure 1 presents the provincial distribution of comprehensive testing coverage, defined as the proportion of pregnant women who reported receiving all three screening tests (HIV, syphilis, and HBV) at any point during pregnancy. The map indicates substantial variation across Indonesia, with several provinces, particularly in Java, Nusa Tenggara, and parts of Kalimantan and Papua, showing coverage exceeding 75%. In contrast, coverage in some provinces, mainly in Central Sumatra and parts of Eastern Indonesia, remain below 45.4%. This situation highlights the interprovincial disparity in access to and utilisation of comprehensive testing services for pregnant women in Indonesia.

Bivariate analyses using simple logistic regression (Table 1) showed that early initiation of ANC was significantly associated with higher odds of completing all three tests (HIV, syphilis, and HBV). Women aged 20 to 34 years and those with higher levels of educational attainment were more likely to achieve complete testing coverage. Conversely, delayed ANC initiation, lower education, and residence in Sumatra, Sulawesi, or Maluku-Papua were associated with lower odds of complete screening. Reproductive characteristics such as parity and age at first pregnancy were also significant, with primiparous and multiparous women demonstrating greater uptake compared with grand multiparas. In addition, health service factors – including receiving ANC from an obstetrician or general practitioner and attending care at public health facilities – were positively associated with complete screening coverage.

In the multivariable logistic regression model (Table 2), the included variables were timing of the first ANC visit, region, maternal education, parity, and type of health facility and were analysed using the enter method. Timing of the first ANC visit remained a strong predictor of complete testing. Women initiating ANC later in pregnancy were 32% less likely to receive the full testing package than those initiating early (AOR = 0.68; 95% CI: 0.53, 0.86; *P* = 0.002). Regional disparities persisted after adjustment. Compared to Java-Bali, women residing in Sumatra (AOR = 0.32; 95% CI: 0.25, 0.42; *P* = 0.001), Sulawesi (AOR = 0.59; 95% CI: 0.45, 0.79; *P* = 0.001), and Maluku-Papua (AOR = 0.47; 95% CI: 0.33, 0.69; *P* = 0.001) had significantly lower odds of receiving comprehensive testing, while coverage in Kalimantan (AOR = 0.74; 95% CI: 0.51, 1.08; *P* = 0.114) and Nusa Tenggara (AOR = 1.03; 95% CI: 0.66, 1.52; *P* = 0.896) was not significantly different from Java-Bali.

## Discussion

Our study highlights that the timing of ANC initiation has a pivotal role in ensuring pregnant women receive comprehensive screening for HIV, syphilis, and HBV. Women who began ANC late in pregnancy had a 32% lower likelihood of receiving the complete screening package compared to those who initiated early (AOR = 0.68; 95% CI: 0.53, 0.86). This underscores the critical window of early ANC for counselling, testing, and timely referrals, essential to EMTCT strategies. Early engagement facilitates these interventions and increases opportunities for follow-up care, adherence monitoring, and prophylactic treatment when indicated. Our findings are consistent with global evidence showing that early ANC initiation extends the timeframe for essential interventions, thereby enhancing the effectiveness of EMTCT programmes (13). For instance, research in Ethiopia demonstrated that delayed ANC substantially reduced access to integrated screening services (14). At the same time, multi-country studies in Sub-Saharan Africa reported that more than half of the pregnant women initiated ANC late, limiting their access to timely maternal and child health interventions (15).

Beyond timing, our results reveal significant regional disparities in the uptake of comprehensive screening. Women residing in Sumatra, Sulawesi, and Maluku-Papua had markedly lower odds of receiving all three tests than those in Java-Bali. These disparities mirror systemic inequities in health infrastructure, workforce distribution, and supply chain robustness across regions. A previous national study showed that women in Java-Bali were over three times more likely to complete four or more ANC visits than those in Papua (16). Additional evidence from Bali, Indonesia, has highlighted challenges in EMTCT implementation, particularly due to limitations in facility readiness and workforce distribution (17). Comparable findings from Uganda further emphasise that the success of integrated maternal screening depends heavily on facility-level readiness, availability of maternal commodities, and effective district-level coordination (18). This body of evidence corroborates our findings and underscores the universal challenge of addressing regional disparities in maternal health service delivery.

Systemic bottlenecks within the health system further constrain the integration of HIV, syphilis, and HBV screening into ANC services. Even when women seek ANC early, inadequate facility readiness, limited workforce capacity, and supply chain gaps can undermine the delivery of comprehensive services (19–22). Addressing these barriers requires investments in infrastructure, consistent supply of maternal commodities, and the adoption of dual or triple rapid diagnostic tests at the first ANC visit (21–24). Such measures would streamline service delivery, reduce missed opportunities for timely screening, and enhance accountability across regions.

Our results suggest two interlinked challenges in the maternal health landscape of Indonesia. First, delayed ANC initiation continues to hinder the uptake of comprehensive screening. Second, regional inequities persist, with women in eastern and outer islands disproportionately underserved. The combination of late pregnancy initiation of ANC and residence outside Java-Bali compounds the risk of missed opportunities for timely diagnosis and intervention. To accelerate progress towards EMTCT of HIV, syphilis, and HBV, policy responses must adopt a dual approach. On the demand side, interventions should promote early ANC initiation through public awareness campaigns and community engagement. On the supply side, investments are needed to strengthen health system readiness by improving facility infrastructure, securing maternal commodity supply, and training healthcare providers. Implementing dual rapid testing for HIV, syphilis, and HBV at the first ANC visit could streamline service delivery and enhance coverage (25, 26).

Strengthening health information systems is equally vital to monitor service delivery and ensure accountability. These findings carry important policy implications for the maternal health programmes of Indonesia. Strengthening demand-side interventions requires sustained community mobilisation and health promotion campaigns to encourage earlier ANC attendance, particularly in rural and underserved areas. At the same time, supply-side improvements should focus on enhancing facility readiness, ensuring the uninterrupted availability of rapid test kits and essential maternal commodities, and building the capacity of frontline health workers. Expanding dual or triple rapid diagnostic tests during the first ANC visit could streamline service delivery and reduce missed opportunities for timely screening (25). Furthermore, integrating socio-economic support mechanisms such as transportation subsidies, conditional cash transfers, or community-based outreach could mitigate the financial and structural barriers disadvantaged women face. By aligning these strategies under a coordinated national framework, Indonesia can accelerate progress towards EMTCT targets and improve equity in maternal and child health outcomes across regions.

### Limitations and Future Research

This observational design of this study limits the ability to establish causal relationships between ANC timing and screening outcomes. Unmeasured confounders, such as facility readiness indicators and patient-level factors like stigma, may influence the observed associations. Additionally, as the data relied on self-reported information, recall and reporting biases may have affected the accuracy of responses. Although the survey was conducted at a national scale, the absence of certain critical variables, such as quality-of-care indicators and psychosocial factors, may limit the generalisability of the findings. Future research should employ longitudinal and mixed-methods approaches to better explore the causal pathways linking ANC timing to screening outcomes. Studies should also evaluate the effectiveness of integrated service delivery models and assess targeted interventions aimed at reducing regional disparities and improving maternal health outcomes.

## Conclusion

Early initiation of ANC is a critical determinant of comprehensive screening for HIV, syphilis, and HBV. However, regional disparities, health system bottlenecks, and socio-economic factors can undermine the effectiveness of early ANC in achieving complete screening coverage. To meet the EMTCT targets of Indonesia, developing dual or triple rapid diagnostics at the first ANC visit and prioritising investments in underserved regions are urgent priorities. Addressing these challenges through integrated policy approaches that enhance both demand and supply aspects of maternal health services remains essential for the successful EMTCT of these infections.

## Figures and Tables

**Table 1:** Maternal, reproductive, and health service factors associated with coverage of HIV, syphilis, and HBV elimination services among pregnant women in Indonesia

Variables	Coverage of HIV, syphilis, and HBV elimination services		Adjusted OR (95% CI)	*P*-value

	Incomplete (*n* = 759) *n* (%)	Complete (*n* = 1,204) *n* (%)		
Timing of the first ANC visit				
Early pregnancy	588 (36.9)	1,002 (63.1)	Ref	0.001
Late pregnancy	171 (45.8)	202 (54.2)	0.68 (0.54, 0.85)	

Demographic variables				

Maternal age				0.053
20 to 34 years	581 (37.5)	967 (62.5)	Ref	
< 20 years and > 34 years	178 (42.9)	237 (57.1)	0.81 (0.65, 1.00)	

Maternal education				
College	183 (34.5)	347 (65.5)	Ref	0.347
Senior high school	287 (36.2)	507 (63.8)	0.91 (0.74, 1.12)	0.003
Junior high school	136 (42.1)	187 (57.9)	0.68 (0.53, 0.88)	0.001
Primary	153 (48.4)	163 (51.6)	0.53 (0.41, 0.69)	

Residence				0.272
Urban	187 (53.2)	165 (46.8)	Ref	
Rural	166 (48.7)	175 (51.3)	1,19 (0.87, 1.61)	

Region				
Java-Bali	136 (26.3)	382 (73.7)	Ref	
Sumatra	321 (52.5)	291 (47.5)	0.28 (0.21, 0.36)	0.001
Kalimantan	54 (32.5)	112 (67.5)	0.72 (0.50, 1.02)	0.066
Sulawesi	136 (38.4)	218 (61.6)	0.61 (0.46, 0.80)	0.001
Nusa Tenggara	40 (26.7)	110 (78.3)	1.02 (0.67, 1.55)	0.918
Maluku and Papua	72 (44.2)	91 (55.8)	0.49 (0.35, 0.68)	0.001

Reproductive variables				

Age at first pregnancy				
20 to 34 years	595 (37.6)	989 (62.4)	Ref	0.046
< 19 years and ≥ 35 years	164 (43.3)	215 (56.7)	0.84 (0.71, 0.99)	

Gravidity				
Multigravida	603 (37.4)	1,008 (62.6)	0.81 (0.64, 1.03)	0.055
Primigravida	68 (44.5)	85 (55.5)	0.79 (0.61, 1.03)	0.072
Grand multigravida	88 (44.2)	111 (55.8)		

Parity				
Multipara	409 (40.9)	591 (59.1)	Ref	
Primipara and nullipara	314 (34.9)	586 (65.1)	1.23 (1.04, 1.46)	0.019
Grand multipara	36 (57.1)	27 (42.8)	0.52 (0.32, 0.86)	0.001

Pregnancy intention				
Intended	721 (38.3)	1,161 (61.7)	Ref	
Unintended	38 (46.9)	43 (53.1)	0.84 (0.65, 1.09)	0.149

Health service variables				

ANC visit with obstetrician				
No	384 (45.1)	467 (54.9)	Ref	0.001
Yes	375 (33.7)	737 (66.3)	1.61 (2.30, 2.00)	

ANC visit with general practitioner				
No	731 (39.3)	1,129 (60.7)	Ref	0.018
Yes	28 (27.2)	75 (72.8)	1.74 (1.10, 2.74)	

ANC visit with midwife				
No	108 (45.5)	146 (57.5)	Ref	0.199
Yes	651	1,058 (61.9)	1.21 (0.90, 1.62)	

ANC visit with others healthcare providers				
No	748 (38.8)	1,182 (61.2)	Ref	0.649
Yes	11 (33.3)	22 (66.7)	1.22 (0.53, 2.81)	

Place of health check-up				
Primary health care	133 (30.8)	298 (69.2)	Ref	
Hospital	74 (30.9)	165 (69.1)	0.99 (0.69, 1.42)	0.981
Private clinic/private practice	357 (43.2)	469 (56.8)	0.57 (0.44, 0.74)	0.001
Integrated health post/village maternity post	195 (41.8)	272 (58.2)	0.64 (0.48, 0.85)	0.002

Data were analysed from the 2023 Indonesian Health Survey

**Table 2:** Multivariate analysis of the association between timing of first antenatal care visit, regional residence, and uptake of maternal HIV, syphilis, and HBV services in Indonesia

Variables	AOR (95% CI)	*P*-value
Timing of the First ANC Visit		
Early pregnancy	ref	
Late pregnancy	0.68 (0.53, 0.86)	0.002

Region		
Java-Bali	ref	
Sumatra	0.32 (0.25, 0.42)	0.001
Kalimantan	0.74 (0.51, 1.08)	0.114
Sulawesi	0.59 (0.45, 0.79)	0.001
Nusa Tenggara	1.03 (0.66, 1.52)	0.896
Maluku and Papua	0.47 (0.33, 0.69)	0.001

AOR = Adjusted odds ratio; Data were analysed from the 2023 Indonesian Health Survey

## References

[1] World Health Organization (2022). Regional Office for South-East Asia. Integrated Regional Action Plan for viral hepatitis, HIV and sexually transmitted infections in South-East Asia, 2022–2026 [Internet].

[2] The Joint United Nations Programme on HIV/AIDS (UNAIDS) (2024). The urgency of now: AIDS at a crossroads. 2024 global AIDS update [Internet].

[3] World Health Organization (2024). Global hepatitis report 2024: action for access in low- and middle-income countries [Internet].

[4] World Health Organization (2023). Global guidance on criteria and processes for validation: elimination of mother-to-child transmission of HIV, syphilis and hepatitis B virus: web annex A: checklist for country preliminary assessment of EMTCT of HIV, syphilis and hepatitis B virus and path to elimination criteria [Internet].

[5] World Health Organization (2019). Dual HIV/syphilis rapid diagnostic tests can be used as the first antenatal care [Internet].

[6] World Health Organization (2022). Sexually transmitted infections (STIs) [Internet].

[7] World Health Organization (2025). Launch of the regional roadmap for elimination of mother-to-child transmission of HIV, syphilis and hepatitis B in the Asia and Pacific region for 2024–2030 [Internet].

[8] UNAIDS (2023). An evaluation of the contribution of the UNAIDS Joint Programme to strengthening HIV and primary health care outcomes [Internet].

[9] Indonesian Ministry of Health (2025). Executive summary of HIV/AIDS and PIMS developments, January to December 2024 [Internet].

[10] Indonesian Ministry of Health (2024). Executive summary of HIV/AIDS and PIMS developments, January to December 2023 [Internet].

[11] Azhali BA, Setiabudi D, Alam A (2023). Evaluating the impact of triple elimination program for mother-to-child transmission of HIV, syphilis, and hepatitis B in Indonesia. Narra J.

[12] Salma (2024). Antenatal testing helps prevent HIV transmission from pregnant mothers to children [Internet].

[13] Mangold JF, Goswami R, Nelson AN, Martinez DR, Fouda GG, Permar SR (2021). Maternal interventions to prevent mother-to-child transmission of HIV: moving beyond antiretroviral therapy. Pediatr Infect Dis J.

[14] Ewunetie AA, Munea AM, Meselu BT, Simeneh MM, Meteku BT (2018). Delay on first antenatal care visit and its associated factors among pregnant women in public health facilities of Debre Markos town, North West Ethiopia. BMC Pregnancy Childbirth.

[15] Dibaba B, Bekena M, Dingeta T, Refisa E, Bekele H, Nigussie S (2024). Late initiation of antenatal care and associated factors among pregnant women attending antenatal clinic at Hiwot Fana Comprehensive Specialized Hospital, Eastern Ethiopia: a cross-sectional study. Front Glob Womens Health.

[16] Laksono AD, Rukmini R, Wulandari RD (2020). Regional disparities in antenatal care utilization in Indonesia. PLoS One.

[17] Armini LN, Setiawati EP, Arisanti N, Hilmanto D (2023). Evaluation of process indicators and challenges of the elimination of mother-to-child transmission of HIV, syphilis, and hepatitis B in Bali Province, Indonesia (2019–2022): a mixed methods study. Trop Med Infect Dis.

[18] Alege JB, Oyore JP, Nanyonga RC, Musoke P, Orago ASS (2025). Barriers and facilitators of integrated hepatitis B, C, and HIV screening among pregnant mothers and newborns attending maternal and newborn clinics in Koboko District, Uganda: a qualitative inquiry of providers’ perspective. BMC Infect Dis.

[19] Kazibwe A, Olal E, Ojok AM, Kigongo JV, Kafumbe H, Niwampeire MP (2025). Facilitators, barriers and service availability for delivering integrated care for the triple elimination of HIV, syphilis and hepatitis B vertical transmission in Uganda: a multi-site explanatory mixed methods study. BMC Health Serv Res.

[20] Suparmi S, Afifah T, Masitoh S, Oktarina O, Sulistiyowati N, Nugraheny E (2023). Socioeconomic difference and adequate antenatal care in Indonesia: evidence from a nationwide household survey. Open Access Maced J Med Sci.

[21] Idris H, Karimah RN, Yulianti A (2025). Urban–rural differences in the incompleteness of antenatal care coverage in Indonesia: a cross-sectional study. Malays Fam Physician.

[22] Wulandari RD, Laksono AD, Rohmah N (2021). Urban–rural disparities of antenatal care in South East Asia: a case study in the Philippines and Indonesia. BMC Public Health.

[23] Shibre G, Zegeye B, Idriss-Wheeler D, Ahinkorah BO, Oladimeji O, Yaya S (2020). Socioeconomic and geographic variations in antenatal care coverage in Angola: further analysis of the 2015 demographic and health survey. BMC Public Health.

[24] Fatiah MS, Purba R, Mollet GCC, Bela SRA (2025). The effect of being faithful, condom use, no drug behavior on the incidence of sexually transmitted infections (STIs) in unmarried men who have sex with men (MSM) in Indonesia. Unnes J Public Health.

[25] Swartzendruber A, Steiner RJ, Adler MR, Kamb ML, Newman LM (2015). Introduction of rapid syphilis testing in antenatal care: a systematic review of the impact on HIV and syphilis testing uptake and coverage. Int J Gynaecol Obstet.

[26] Wulandari RD, Laksono AD, Allo Bela SR, Fatiah MS, Rohmah N, Widya Sukoco NE (2025). Determining policy targets for reducing the number of stunted Papuan children under five years old in Indonesia: a secondary data analysis of the 2022 Indonesian National Nutritional Status Survey. Malays J Med Sci.

